# Imaging and Non-Imaging Approaches for the Diagnosis and Monitoring of Necrotizing Enterocolitis—What Lies Ahead?

**DOI:** 10.3390/children13060787

**Published:** 2026-06-05

**Authors:** Indrani Bhattacharjee, Catalina Le Cacheux, Eric B. Ortigoza, Jonathan Dillman, Sherwin S. Chan, Alain Cuna

**Affiliations:** 1 Department of Pediatrics, Tufts University School of Medicine, Boston, MA 02111, USA; indrani.bhattacharjee@tuftsmedicine.org; 2 Department of Radiology, Children’s Mercy Kansas City, Kansas City, MO 64108, USA; clecacheux@cmh.edu (C.L.C.); sschan@cmh.edu (S.S.C.); 3 School of Medicine, University of Missouri-Kansas City, Kansas City, MO 64108, USA; 4 Division of Neonatal-Perinatal Medicine, Department of Pediatrics, UT Southwestern Medical Center, Dallas, TX 75390, USA; eric.ortigoza@utsouthwestern.edu; 5 Department of Radiology, Cincinnati Children’s Hospital Medical Center, Cincinnati, OH 45229, USA; jonathan.dillman@cchmc.org; 6 Division of Neonatology, Department of Pediatrics, Children’s Mercy Kansas City, Kansas City, MO 64108, USA

**Keywords:** necrotizing enterocolitis, ultrasound, imaging, diagnostics, prematurity, neonate

## Abstract

**Highlights:**

**What are the main findings?**

• Current imaging for necrotizing enterocolitis, centered on abdominal radiography and bowel ultrasound, primarily detects and monitors established intestinal injury.
• Emerging imaging modalities aim to characterize microvascular perfusion, oxygenation, and microstructural changes that may enable earlier detection and diagnosis.
• Complementary physiologic monitoring approaches may enable early risk stratification by detecting abnormal patterns in intestinal perfusion, oxygenation, and motility before clinical disease develops.

**What are the implications of the main findings?**

• Necrotizing enterocolitis evaluation is shifting from detection of late disease to earlier identification of intestinal vulnerability.
• Artificial intelligence may integrate imaging and physiologic data to improve diagnostic accuracy and risk stratification.
• Neonatologist-performed bowel ultrasound may expand access to bedside imaging and enable more timely physiologic assessment in neonatal intensive care settings.

**Abstract:**

Necrotizing enterocolitis (NEC) remains one of the most serious gastrointestinal emergencies in preterm infants, and imaging plays a central role in diagnosis and clinical management. Historically, evaluation has relied primarily on abdominal radiography, which remains widely available and embedded in established diagnostic frameworks. However, the hallmark radiographic signs of NEC (i.e., pneumatosis intestinalis, portal venous gas, and free air) reflect relatively advanced manifestations of intestinal injury that indicate established mucosal disruption or transmural necrosis. Bowel ultrasound has increasingly complemented radiography by enabling real-time assessment of bowel wall integrity, perfusion, motility, and intra-abdominal fluid, providing physiologic information that may refine clinical interpretation and monitoring of disease progression. Expanding use of neonatologist-performed bowel ultrasound may further improve access to bedside intestinal imaging and facilitate more timely evaluation in neonatal intensive care settings. In parallel, emerging imaging technologies seek to extend the capabilities of conventional imaging by interrogating biologic processes that underlie intestinal injury. Modalities such as contrast-enhanced ultrasound, ultra-high-frequency ultrasound, and photoacoustic imaging offer the potential to characterize bowel microvascular perfusion, tissue oxygenation, and microstructural changes that may precede overt radiographic abnormalities. Complementary physiologic monitoring approaches are also being explored to identify infants at risk before clinical disease develops. Techniques including superior mesenteric artery Doppler, near-infrared spectroscopy, bowel acoustic monitoring, and electrogastrography aim to detect early alterations in intestinal perfusion, oxygenation, and motility. In addition, artificial intelligence applied to imaging and physiologic data may enhance pattern recognition, risk stratification, and clinical decision support. Together, these advances suggest that NEC evaluation is evolving from a paradigm focused on detecting late structural injury toward integrated approaches capable of identifying intestinal vulnerability earlier and monitoring disease more precisely.

## 1. Introduction

Necrotizing enterocolitis (NEC) is a severe gastrointestinal disease of prematurity characterized by intestinal inflammation and varying degrees of bowel necrosis, with potential progression to perforation. NEC remains a major cause of morbidity and mortality among preterm and very low-birth-weight infants, with an estimated incidence of 7–12% and mortality rates ranging from 20% to over 50% in surgical cases [1,2,3]. The pathophysiology of NEC is multifactorial and incompletely understood, involving intestinal immaturity, dysregulated inflammatory responses, microbial dysbiosis, and impaired intestinal perfusion.

Early clinical manifestations of NEC are nonspecific and overlap with other neonatal conditions such as sepsis, spontaneous intestinal perforation, and benign feeding intolerance of prematurity. Common clinical findings include feeding intolerance, abdominal distention, emesis, bloody stools, and systemic signs of illness. Laboratory abnormalities may include thrombocytopenia, metabolic acidosis, leukopenia or leukocytosis, and elevated inflammatory markers. Overall, these clinical and laboratory findings are neither sensitive nor specific for NEC. In the absence of rigorously validated and widely used clinical biomarkers, imaging has become central to NEC diagnosis, guiding both clinical staging and therapeutic decision-making [4,5].

Abdominal radiography (AXR) remains the most widely used modality and forms the backbone of established diagnostic frameworks. Classic radiographic signs—including pneumatosis intestinalis, portal venous gas, and pneumoperitoneum—support diagnosis of NEC and guide medical and surgical interventions [6]. These findings are incorporated into commonly used classification systems, including the Modified Bell staging criteria and the Vermont Oxford Network criteria [7,8]. These classification systems provide standardized definitions for NEC that combine clinical, laboratory, and radiographic features, facilitating diagnosis in clinical research and quality improvement initiatives, as well as stratifying disease severity and guiding clinical management. In recent years, bowel ultrasound (BUS) has emerged as an important adjunct to AXR, offering real-time, radiation-free imaging of intestinal features including wall thickness, echogenicity, peristalsis, and mesenteric blood flow [9]. Growing evidence suggests that BUS may detect early inflammatory and perfusion changes to the bowel wall before radiographic abnormalities become apparent [10].

Beyond AXR and BUS, several emerging imaging modalities seek to provide additional physiologic and microvascular characterization for differentiating intestinal injury of NEC from other conditions. Contrast-enhanced ultrasound (CEUS), ultra-high-frequency ultrasound (UHFUS), and photoacoustic imaging (PAI) aim to provide more granular characterization of intestinal microvascular perfusion, wall architecture, and tissue oxygenation [11]. In addition, tools like superior mesenteric artery (SMA) Doppler, near-infrared spectroscopy (NIRS), bowel acoustic analysis, and electrogastrography (EGG) seek to capture dynamic changes in intestinal blood flow and motility that may contribute physiologic risk stratification and disease monitoring of infants at higher risk for NEC. In parallel, advances in neonatologist-performed bowel ultrasound and artificial intelligence (AI) applications may further enhance NEC imaging by improving accessibility, standardized interpretation, and risk prediction.

The aim of this narrative review is to synthesize current and emerging imaging and non-imaging physiologic approaches for the diagnosis and monitoring of NEC. The literature discussed was identified based on relevance to clinical practice and emerging translational impact, with the goal of appraising strengths and limitations and highlighting future directions that may improve early detection, risk stratification, and clinical decision-making.

## 2. Current Imaging for Diagnosis and Monitoring of NEC

### 2.1. Abdominal Radiograph (AXR)

AXR has served as the cornerstone of imaging for NEC since its initial description in 1964 [12]. It remains the most widely used imaging modality due to its broad availability, rapid bedside acquisition, and familiarity among clinicians. Importantly, AXR findings are embedded within established diagnostic frameworks, such as the modified Bell staging and the Vermont Oxford Network criteria [13]. As such, AXR continues to anchor diagnostic classification and treatment algorithms for NEC (Table 1).

#### 2.1.1. Role of AXR in NEC Diagnosis and Disease Monitoring

**Figure 1:**
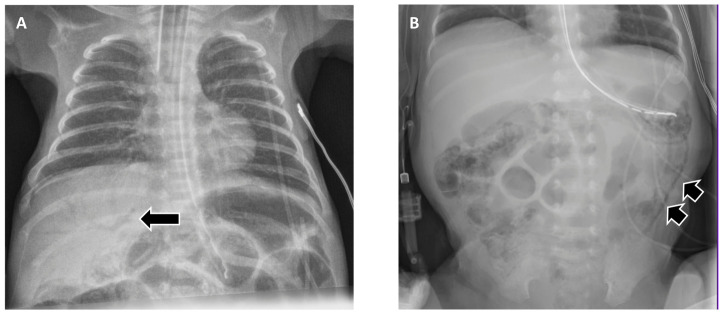
Classic abdominal radiographic findings in necrotizing enterocolitis. (**A**) Supine abdominal radiograph demonstrating portal venous gas (black arrow), seen as branching, linear lucencies within the hepatic parenchyma, reflecting intravascular gas within the portal venous system. (**B**) Supine abdominal radiograph demonstrating pneumatosis intestinalis, with linear “train-track” intramural lucencies along the bowel wall (arrowhead). These radiographic findings typically reflect mucosal injury with translocation of gas-producing bacteria into the bowel wall and portal venous system and are associated with advanced intestinal injury in NEC.

The hallmark radiographic features for diagnosing NEC include (1) pneumatosis intestinalis—intramural gas within the bowel wall; and (2) portal venous gas, seen as branching lucencies overlying the liver. In the appropriate clinical context, these findings are considered pathognomonic for NEC (Figure 1) [14]. In addition, pneumoperitoneum—free intraperitoneal air indicating perforation—indicates advanced disease and constitutes a surgical emergency. In addition to these classic signs, several indirect or nonspecific radiographic features may raise concern for evolving NEC, including dilated bowel loops, asymmetric or fixed bowel gas patterns, a gasless abdomen, and bowel wall thickening [15]. However, these findings lack specificity for NEC and must be interpreted cautiously in conjunction with the clinical picture.

Beyond initial diagnosis, AXR plays an important role in monitoring disease progression and guiding management. Serial radiographs serve as a surrogate marker of disease trajectory, particularly in infants with equivocal or evolving clinical signs. Interval imaging serves to assess changes over time, including the development or resolution of pneumatosis intestinalis or portal venous gas, and the emergence of pneumoperitoneum. These evolving findings may prompt escalation or de-escalation of care, depending on the overall clinical picture [16].

#### 2.1.2. Limitations of AXR

Despite its central role, AXR has important limitations. First, its sensitivity for early NEC is low. In a retrospective cohort of 80 infants with surgically confirmed NEC or perforation, Tam et al. demonstrated that traditional radiographic signs were highly specific (92–100%) but poorly sensitive [17]. Pneumatosis intestinalis and portal venous gas were present in only 44% and 13% of cases, respectively. Even pneumoperitoneum, often considered an absolute surgical indication, was detected in just 52% of cases. Overall negative predictive values ranged from 26–61%, underscoring that absence of radiographic findings does not reliably rule out NEC or perforation.

Second, AXR is constrained by substantial interobserver variability. In a prospective study of 40 radiographs interpreted twice by 12 observers of varying experience levels (2 pediatric radiologists, 4 attending neonatologists, 3 neonatal fellows, and 3 pediatric residents), Rehan et al. demonstrated only fair interobserver agreement for key radiographic findings, including pneumatosis intestinalis (κ ≈ 0.24), portal venous gas (κ ≈ 0.28), and pneumoperitoneum (κ ≈ 0.32) [18]. In a larger cohort of 297 radiographs interpreted independently by three expert pediatric radiologists, Di Napoli et al. also found poor agreement for most radiographic signs, including pneumatosis intestinalis (κ = 0.13–0.29) and portal venous gas (κ = 0.10) [19]. These findings underscore variability in radiographic interpretation, contributing to inconsistencies in NEC diagnosis and clinical decision-making [16,20,21].

Third, AXR necessitates exposure to ionizing radiation. In a large retrospective cohort of 1045 premature infants ≤ 32 weeks’ gestation, Khattab et al. demonstrated that infants with NEC had a median cumulative effective dose of 1228 µSv compared with 136 µSv in those without NEC, and NEC independently predicted an increase of over 1100 µSv after multivariable adjustment [22]. These findings underscore that, while individual AXRs carry modest radiation dose, infants with suspected or evolving NEC often undergo serial examinations, resulting in substantial cumulative exposure in this radiosensitive population.

Lastly, AXR is limited to static, 2D anatomic snapshots with limited physiological insight [23,24]. AXR cannot assess bowel perfusion, motility, or characterize peritoneal fluid, and it cannot reliably differentiate simple from inflammatory ascites. These constraints, coupled with the need for repeated imaging, have stimulated increasing interest in radiation-free modalities that provide real-time functional assessment.

### 2.2. Bowel Ultrasound (BUS)

**Figure 2:**
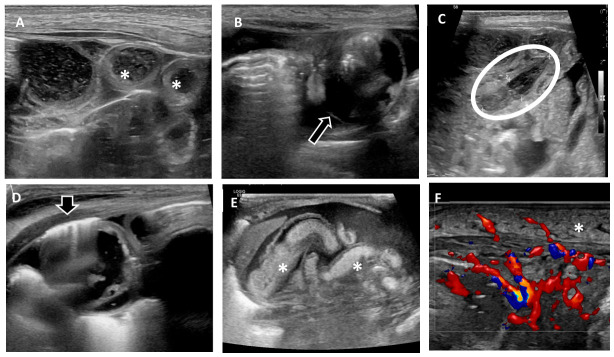
Ultrasound findings associated with necrotizing enterocolitis. (**A**) Thickened bowel loops (asterisks), reflecting bowel wall edema and inflammation. (**B**) Thinned bowel wall (arrow), concerning for bowel wall ischemia and increased risk of perforation. (**C**) Complex intra-abdominal fluid collection (oval), containing internal echogenic debris, suggestive of inflammatory or infected ascites. (**D**) Pneumoperitoneum (arrowhead), identified by echogenic free intraperitoneal gas with associated reverberation artifact, suggestive of bowel perforation. (**E**) Hyperechoic luminal contents (asterisks), characterized by diffusely echogenic material within the bowel lumen, suggestive of intraluminal hemorrhage, mucin, sloughed intestinal tissue, and inflammatory debris. (**F**) Abdominal wall edema and hyperemia, characterized by thickening and increased echogenicity of the abdominal wall (asterisk) with increased Doppler flow, suggestive of abdominal wall inflammation associated with NEC.

Bowel ultrasound (BUS) has become an increasingly important imaging modality in suspected or confirmed NEC because it provides real-time assessment of bowel wall integrity, perfusion, motility, and peritoneal fluid—domains that are incompletely characterized by AXR (Table 1). BUS can image anatomic pathology (e.g., pneumatosis intestinalis, portal venous gas, free air, ascites/complex fluid collections) while adding physiologic information (bowel wall Doppler perfusion, peristalsis) that can refine risk stratification and surgical decision-making [25,26,27,28] (Figure 2). Newer BUS findings have also been proposed that correlate to patient outcomes [29].

#### 2.2.1. Evidence Synthesis and Diagnostic Performance

Multiple meta-analyses show that many individual BUS findings are highly specific and modestly sensitive for NEC diagnosis when Bell staging is used as the reference standard. The 2018 diagnostic meta-analysis by Cuna et al. showed that classic findings such as portal venous gas, pneumatosis intestinalis, and free air demonstrated high specificities (generally >0.9) but lower sensitivities, supporting BUS as a confirmatory test rather than a stand-alone rule-out test [30]. Complementing diagnostic accuracy, prognostic meta-analysis has linked several sonographic features to severe outcomes (surgery or death), notably complex ascites and focal fluid collections, along with absent peristalsis and absent perfusion—findings that may indicate transmural injury, impending perforation or a perforated bowel. Importantly, some commonly recognized signs (e.g., pneumatosis intestinalis, portal venous gas) may not independently predict surgery or death in pooled analyses, underscoring the incremental value of integrating “high-risk” physiologic and fluid findings into clinical interpretation [31,32]. In addition, it is important to recognize that the patchy and segmental involvement of the intestine in NEC may lead to under-recognition of affected areas if they are not captured during the BUS examination.

#### 2.2.2. Standardization and Implementation

Persistent barriers to broad adoption of BUS include a steep learning curve and the lack of clinical guidelines and standardized protocols. BUS is not routinely taught during radiology residency, and there is a persistent perception that the bowel is difficult—or even impossible—to evaluate with ultrasound. Some of the learning challenges can be mitigated using excellent references that specifically address techniques and standard protocols for BUS acquisition and interpretation [25,33,34]. A recent American College of Radiology Appropriateness guideline on pediatric abdominal pain suggests BUS is a “may be appropriate” exam in the setting of NEC [35]. Additionally, a recent paper showed that tiered BUS exams can be performed with comprehensive exams performed during the day and abbreviated exams to answer critical time sensitive questions in the off hours [36]. Recently, Hegedus et al. reported a standardized universal adjunct abdominal ultrasound protocol performed at the time of initial NEC evaluation in a level IV neonatal intensive care unit (NICU), demonstrating high feasibility (BUS obtained in 96% of NEC evaluations after guideline implementation) and frequent detection of sonographic abnormalities, including cases with initially unremarkable AXR [37]. Notably, BUS findings led to an increased modified Bell stage in more than half of cases compared with AXR alone, illustrating how standardized BUS may reclassify disease severity early in the evaluation pathway.

#### 2.2.3. Emerging Outcomes Data

Beyond diagnostic/prognostic associations, outcomes-oriented evidence is beginning to emerge. In a recent prospective diagnostic trial preprint, Cuna and colleagues compared AXR alone versus AXR plus BUS in infants evaluated for suspected NEC and reported that adding BUS shortened time to full enteral feeds among infants with prolonged diagnostic evaluations, without an observed increase in adverse events. This strengthens the argument that BUS can improve diagnostic confidence and potentially accelerate recovery trajectories in diagnostically uncertain cases [38,39].

### 2.3. Neonatologist-Performed Bowel Ultrasound

Traditionally performed by radiologists, neonatologist-performed bowel ultrasound has now become increasingly feasible for neonatologists trained in point-of-care ultrasound (POCUS) [11], especially as evidence accumulates for its diagnostic accuracy in NEC [40]. Neonatologist-performed BUS enables immediate assessment of bowel wall characteristics, peristalsis, perfusion, and free fluid—features often critical in differentiating NEC from sepsis, benign feeding intolerance, or continuous positive airway pressure (CPAP)-related distention [41].

#### 2.3.1. Feasibility and Early Studies

The feasibility of neonatologist-performed BUS is supported by several case reports demonstrating the use of POCUS by emergency medicine physicians for diagnosing NEC, highlighting that POCUS can be performed reliably, even by non-radiologists [42,43,44]. In another study, neonatologists were trained to perform point-of-care BUS using a standardized scanning protocol developed jointly with pediatric radiology [45]. For the first 12 months, all BUS exams performed by neonatologists were reviewed and formally reported by a radiologist, and in the following 12 months neonatologists independently performed and interpreted the scans, consulting radiology only for technically difficult cases. The study demonstrated that neonatologists could reliably acquire and interpret BUS images, improving diagnostic confidence and guiding management decisions including surgical referral.

#### 2.3.2. Global Experiences with Neonatologist-Performed BUS

Australia provides some of the earliest foundational experience with neonatologist-performed BUS. Initially focused on cranial and cardiac imaging, neonatal training pathways in Australia have since expanded to include BUS for NEC evaluation [46]. A report of their experience demonstrated that with structured curricula, supervised scanning, and standardized techniques, neonatologists can acquire BUS skills to differentiate normal bowel from abnormal NEC findings and differentiate NEC from NEC mimics such as midgut volvulus and CPAP-belly syndrome [47].

In Europe, structured neonatal POCUS protocols such as Sonographic Assessment of life-threatening Emergencies—Revised (SAFE-R) and Crashing Neonate Protocol (CNP) were created for evaluating critically ill neonates [48,49]. These protocols both endorse neonatologist-performed abdominal POCUS as part of screening for acute abdominal pathology like NEC. Moreover, the European Society of Paediatric and Neonatal Intensive Care (ESPINIC) POCUS Working Group has since released international evidence-based guidelines that explicitly recommend neonatologist-performed POCUS as a helpful tool for detecting signs of NEC in unstable or deteriorating neonates [50].

Within the United States, POCUS adoption in the NICU is also increasing. In 2022, the American Academy of Pediatrics released a technical report that describes the many potential applications of POCUS in the NICU, including abdominal POCUS for assessing bowel ischemia and NEC [51]. A study of a large academic NICU in California demonstrated successful implementation of a bedside POCUS program—which included abdominal applications such as bladder assessment and ascites evaluation—further supporting the feasibility of implementing NICU POCUS programs and the role of abdominal ultrasound in neonatal care [52].

Collectively, these experiences across multiple regions demonstrate that neonatologist-performed BUS is feasible to implement, requires minimal additional equipment beyond existing POCUS infrastructure, and can be readily integrated into clinical workflows [40].

#### 2.3.3. Limitations and Future Directions of Neonatologist-Performed BUS

Despite these advantages, barriers to widespread adoption remain. These include variability in training access, differences in equipment availability and image quality (particularly between POCUS and radiology-grade systems), institutional resistance to non-radiologist imaging, and lack of credentialing pathways in some regions [50,51]. Nonetheless, emerging collaborative models—where radiologists provide oversight or quality control while neonatologists perform frontline scanning—are proving effective [45]. As with cardiac and lung POCUS, the standardization of BUS for neonatologists will require multicenter guidelines, credentialing frameworks, and real-world outcome data [47].

## 3. Emerging and Advanced Imaging Techniques

### 3.1. Contrast-Enhanced Ultrasound (CEUS)

Contrast-enhanced ultrasound (CEUS) is a sonographic technique that uses intravenously administered microbubble contrast agents, which remain confined to the intravascular space and enable real-time assessment of tissue perfusion. The microbubbles consist of a sulfur hexafluoride gas core stabilized by a phospholipid shell; the gas core is eliminated via the lungs, while the shell components are cleared by the liver [53]. CEUS allows dynamic assessment of both macro- and microcirculation, making it possible to detect subtle perfusion abnormalities that may occur before structural bowel injury or ischemia becomes apparent (Table 2).

#### 3.1.1. Potential Applications in NEC

CEUS is radiation-free, can be performed at the bedside, and the contrast agents are not nephrotoxic, which makes CEUS particularly well suited for neonatal imaging [54]. In NEC, CEUS offers the ability to perform quantitative assessment of bowel wall perfusion, which may allow detection of intestinal ischemia before conventional radiographic or sonographic signs appear [55]. By characterizing perfusion patterns in real time, CEUS may contribute to risk stratification, identifying infants at higher risk of progression to severe NEC. CEUS may be particularly valuable in clinical scenarios where Doppler assessment of bowel perfusion is limited or unreliable, such as in infants receiving high-frequency oscillatory ventilation [56].

#### 3.1.2. Limitations and Challenges

Despite its promise, CEUS remains limited in neonatal NEC evaluation [57]. Clinical adoption has been slow, particularly in the United States, largely because the use of ultrasound contrast agents in BUS is off label. Technical expertise, standardization of perfusion metrics, and regulatory considerations also remain important barriers to widespread implementation. Furthermore, there is a lack of large prospective studies validating CEUS for NEC prediction or outcome stratification. Currently, bowel CEUS is performed in only a limited number of pediatric hospitals and is used primarily as a troubleshooting tool. It is typically reserved for situations in which patient-specific factors, ventilatory support, or clinical presentation make it difficult to confidently determine whether an abnormal bowel loop seen on B-mode or color Doppler imaging truly represents ischemia [56].

### 3.2. Ultra-High-Frequency Ultrasound (UHFUS)

Ultra-high-frequency ultrasound (UHFUS) is a rapidly evolving imaging modality in neonatal care, offering unprecedented spatial resolution in the 30–70 MHz range—significantly higher than conventional transducers [58]. This improved resolution enables detailed visualization of bowel wall layers, microvasculature, and early inflammatory changes in neonates with suspected NEC (Table 2).

#### 3.2.1. Feasibility of UHFUS

While UHFUS has been traditionally utilized in dermatologic and vascular imaging, its application in neonatal intestinal pathology is gaining traction. Recent feasibility studies in preterm animal models and small clinical series suggest that UHFUS can distinguish between bowel wall edema, necrosis, and hyperemia with greater precision than conventional ultrasound. For example, Hawez et al. demonstrated that UHFUS provides high-resolution delineation of bowel wall microanatomy, including mucosa and submucosa, with strong correlation to histological measurements in Hirschsprung’s disease [59]. Although their study focused on resected surgical samples, the findings underscore UHFUS’s potential to detect early bowel wall changes relevant to conditions like NEC. Jacobsen et al. conducted one of the first in-human studies applying UHFUS (48–70 MHz) to neonatal bowel imaging, including a preterm infant with confirmed NEC [58]. This study demonstrated that UHFUS identified a twofold increase in bowel wall thickness and a fivefold increase in thickness of the visceral peritoneal layer adjacent to affected bowel loops, a finding consistent with serosal inflammation in NEC. Moreover, while conventional grayscale imaging can typically delineate all five bowel histoanatomical wall layers in normal bowel, the authors found that UHFUS could detect more subtle mural changes that are often missed by conventional transducers in NEC, highlighting its superior spatial resolution. While this was a feasibility study with limited longitudinal data, the authors proposed that UHFUS may be valuable in monitoring disease progression and response to treatment in NEC, given its ability to capture microstructural changes in real time without radiation exposure [58].

#### 3.2.2. Limitations and Future Directions of UHFUS

Due to reduced depth penetration, UHFUS is currently best suited for superficial bowel loops and may be challenging in larger infants [11]. Equipment availability and operator training also pose barriers to widespread use. Future integration of UHFUS with color Doppler, CEUS, or even AI-based segmentation tools could enhance its diagnostic utility. Standardized protocols and multicenter studies would also be needed to validate the role of UHFUS alongside other modalities.

### 3.3. Photoacoustic Imaging (PAI)

Photoacoustic imaging (PAI) is a hybrid optical–ultrasound modality that enables noninvasive assessment of tissue perfusion and oxygenation [60]. By delivering pulsed laser light and detecting ultrasound waves generated from light-absorbing chromophores such as hemoglobin, PAI provides quantitative information on microvascular blood volume and oxygen saturation. Unlike conventional ultrasound, which primarily reflects structural differences, PAI offers functional insight into tissue hypoxia and microvascular integrity (Table 2).

#### 3.3.1. Feasibility of PAI in NEC

Given that intestinal ischemia and microvascular dysfunction are important contributors to NEC pathogenesis, PAI has emerged as a mechanistically compelling investigational tool. In a multi-stressor neonatal rat model of NEC, Weis et al. demonstrated that multispectral PAI detected significant reductions in intestinal tissue oxygen saturation in NEC pups compared with controls, with changes evident at both early and established stages of injury [61]. These photoacoustic measures of intestinal oxygenation correlated with histologic severity of injury, providing proof-of-concept that PAI-derived physiologic parameters may serve as quantitative biomarkers of NEC. In a prospective pilot study of children with suspected inflammatory bowel disease, Regensburger et al. found that hemoglobin parameters from multispectral optoacoustic tomography, a tomographic implementation of PAI, correlated with endoscopic and clinical measures of disease activity [62]. Together, these findings provide proof-of-principle that transabdominal PAI can detect inflammatory vascular changes in the intestine that correlate with disease.

#### 3.3.2. Limitations of PAI

Despite these promising preclinical data, translation to human neonatal care remains early. Motion artifact, abdominal distention, and gas-filled loops may impair optical and acoustic signal acquisition [63]. Furthermore, current PAI systems require specialized laser-equipped platforms mostly used in research and would necessitate rigorous safety validation prior to clinical deployment, especially in preterm infants. Thus, while PAI offers a potential shift from structural to functional characterization of intestinal injury, further validation in translational and clinical studies is needed.

## 4. Prediction of NEC—Imaging and Non-Imaging Modalities

### 4.1. Doppler Ultrasound of the Superior Mesenteric Artery (SMA)

Because the superior mesenteric artery (SMA) is the main blood supply to the midgut—and NEC is strongly associated with impaired perfusion and ischemia—Doppler assessment of SMA blood-flow velocity patterns has been investigated as a noninvasive method to identify infants with compromised mesenteric blood-flow reserve and increased risk for NEC (Table 3) [64].

#### 4.1.1. Evidence for NEC Prediction

SMA Doppler quantifies blood flow velocity waveforms and derived indices (e.g., peak systolic velocity, end-diastolic velocity, resistive index, and pulsatility index) that can reflect downstream vascular resistance and perfusion patterns. A nested prospective study of very-low birth weight preterm infants who underwent SMA Doppler within the first 12 h (before first feeding) demonstrated that certain Doppler parameters differed between infants who later developed NEC (stage II–III) and controls, supporting the concept that early perfusion patterns may carry predictive information [65]. While several indices (including RI and PI) were elevated, only the systeole/diastole ratio remained independently associated with NEC, with high specificity but modest sensitivity—suggesting potential utility for identifying a high-risk subgroup rather than serving as a stand-alone screening test.

However, the predictive ability of SMA Doppler does not seem to be consistent across all high-risk groups. In preterm small-for-gestational age infants with abnormal antenatal umbilical artery Dopplers (i.e., absent or reversed end-diastolic flow), postnatal SMA measurements on day 1 and day 5 had poor predictive value for NEC or feeding intolerance [66]. This suggests that abnormal SMA Doppler findings may not be predictive of NEC in growth-restricted infants with altered fetal hemodynamics.

#### 4.1.2. Challenges and Limitations of SMA Doppler

One challenge is that SMA Doppler measures velocity rather than direct tissue oxygenation, and its measurements are sensitive to technical factors such as probe positioning and ultrasound settings, including pulse repetition frequency (PRF)-related aliasing, which occurs when true blood-flow velocities exceed the Doppler sampling limit and can lead to misestimation of flow indices. These methodological constraints are critical when interpreting studies that claim predictive value, because early SMA Doppler differences may reflect technical variability rather than true NEC risk [64]. A second challenge is that multiple neonatal factors can shift SMA velocities independent of NEC risk. For example, in very-low-birth-weight infants studied within the first 48 h, small-for-gestational age status, maternal preeclampsia, antenatal absent/reversed end-diastolic flow, and hemodynamically significant patent ductus arteriosus have all been associated with differences in end-diastolic velocity and resistive index, illustrating how “baseline perfusion” varies by clinical context [67]. A third challenge is the diagnostic ambiguity surrounding NEC, especially when classic radiographic findings are uncertain or not uniformly present. Such diagnostic ambiguity in studies can inflate or suppress the apparent predictive performance of Doppler indices in observational datasets [64].

#### 4.1.3. Future Directions

Despite mixed predictive performance, SMA Doppler continues to be explored as a potential bedside physiologic assessment tool because it can be performed serially without radiation and provides real-time information on mesenteric blood-flow patterns [64]. Interpretation of SMA Doppler is context dependent and influenced by multiple confounding factors. Its utility appears strongest when (a) acquired under standardized conditions (e.g., before first feeding, stable hemodynamics), (b) interpreted with awareness of confounders (small for gestational age, patent ductus arteriosus, antenatal Dopplers, ventilatory support), and (c) combined with other NEC risk markers. Future work includes integrating SMA Doppler within the broader imaging framework used for NEC evaluation, complementing radiographs and ultrasound by providing focused assessment of mesenteric perfusion. Interpretation of Doppler findings must also account for baseline physiologic variation and longitudinal changes. Tracking trends in Doppler values and integrating them with the infant’s overall clinical condition are often more informative than relying on a single Doppler measurement or cutoff value [66].

### 4.2. Near-Infrared Spectroscopy (NIRS)

#### 4.2.1. Overview of Splanchnic NIRS

Near-infrared spectroscopy (NIRS) uses differential absorption of near-infrared light by oxygenated and deoxygenated hemoglobin to provide continuous, non-invasive monitoring of regional tissue oxygen saturation (rSO_2_). When applied to the abdomen, NIRS measures splanchnic rSO_2_, which can serve as a bedside surrogate of the balance between intestinal oxygen delivery and consumption and may complement episodic imaging in infants with suspected NEC. Studies have reviewed the potential of abdominal NIRS as a noninvasive bedside physiologic tool under evaluation for early identification and risk stratification within NEC pathways [54,68].

#### 4.2.2. Evidence Base for Splanchnic NIRS in NEC

Prospective observational data in extremely preterm infants support an association between low early splanchnic oxygenation and subsequent NEC. In infants born <28 weeks’ gestation who were predominantly receiving continuous enteral feeding, lower splanchnic rSO_2_ during the first postnatal week was associated with later NEC (Bell Stage ≥ II), with a commonly cited threshold of mean splanchnic rSO_2_ < 30% [69]. A subsequent cohort study evaluated the clinical usefulness of this cutoff and examined its test characteristics for NEC prediction. Although a mean splanchnic rSO_2_ < 30% was associated with increased NEC risk, the positive predictive value was modest and the negative predictive value was high, suggesting greater utility for identifying infants at lower risk than for definitively ruling in disease [70].

Earlier work also demonstrated that abdominal NIRS values were lower and more variable in preterm infants who developed NEC, supporting the concept that impaired or unstable intestinal oxygenation may precede overt disease [71]. In high-risk populations, such as neonates undergoing congenital heart surgery, postoperative splanchnic NIRS patterns have also been associated with subsequent intestinal ischemia and necrosis, supporting its role as an adjunct for risk stratification in infants with single-ventricle physiology [72]. In symptomatic infants, emerging evidence suggests NIRS may be most informative for assessment of disease severity and trajectory in NEC. A retrospective study of preterm infants with clinical concern for NEC found that a high splanchnic–cerebral oxygenation ratio and reduced splanchnic rSO_2_ variability may serve as potential diagnostic adjuncts for diagnosing NEC [73]. A prospective study further demonstrated that multisite NIRS measures (cerebral, liver, and infraumbilical rSO_2_) could help distinguish uncomplicated from complicated NEC, highlighting the potential role of NIRS patterns for risk stratification of infants with NEC [74].

#### 4.2.3. Limitations and Ongoing Research

Despite strong biologic plausibility, not all studies evaluating abdominal NIRS for NEC have demonstrated consistent value. In one study of preterm neonates presenting with acute gastrointestinal symptoms, abdominal and cerebral NIRS obtained at symptom onset did not reliably distinguish NEC from other gastrointestinal pathology, highlighting the variability of NIRS signals and reinforcing that these metrics remain investigational [75]. Several factors contribute to these limitations, including interpatient variability, differences in sensor type and placement, motion artifact, and the impact of bowel gas or abdominal wall characteristics, all of which reduce confidence in single absolute cutoffs across NICUs. Recent computational modeling work using finite element modeling has demonstrated that abdominal tissue composition, probe geometry, and source-detector spacing significantly influence depth sensitivity and signal contamination in infant splanchnic oximetry. These findings highlight the technical complexity of obtaining reliable splanchnic measurements and underscore the importance of sensor design optimization and standardized acquisition protocols to strengthen the clinical utility of NIRS in NEC evaluation [76].

### 4.3. Bowel Acoustics

Acoustic analysis of bowel sounds offers a noninvasive method to characterize intestinal motility and functional gut activity. Because NEC is preceded by disruptions in motility, acoustic monitoring may provide early physiologic signals before overt clinical deterioration (Table 3).

#### 4.3.1. Feasibility of Bowel Acoustics in Preterm Infants

Digital auscultation and automated bowel sound analysis attempt to quantify intestinal motility through objective acoustic features such as event rate, amplitude, and spectral characteristics. Neonatal feasibility has been demonstrated using a long-term acoustic monitoring system in term infants, showing that bowel sounds can be continuously captured and quantitatively characterized over extended monitoring periods [77]. Additional work using a prototype electronic stethoscope system further confirmed the feasibility of prolonged neonatal bowel sound recording using an adhesive bedside interphase [78]. Importantly, feasibility has also been demonstrated in preterm infants. In a multimodal physiologic study incorporating abdominal NIRS, electrogastrography, and acoustic monitoring, bowel sounds were successfully recorded longitudinally in preterm infants, supporting the practicality of bedside acoustic acquisition in this population [79]. Although this study did not use artificial intelligence-based analysis, it established technical feasibility and signal stability in premature infants.

#### 4.3.2. Potential in NEC

Direct evidence that bowel acoustics predict NEC remains limited. However, because bowel sound features vary with feeding exposure and gastrointestinal maturation [77,79], continuous acoustic monitoring may detect evolving dysmotility or altered gut activity. Such physiological changes could serve as a screening signal to identify infants who merit confirmatory imaging.

Integrating acoustic data with ultrasound is conceptually attractive. Ultrasound provides direct visualization of peristalsis, bowel wall characteristics, and perfusion patterns, offering an anatomic and hemodynamic complement to the functional information derived from bowel acoustics. However, in a prospective neonatal study comparing simultaneous bowel sound recordings with real-time ultrasound assessment of peristalsis in well neonates, agreement between acoustic and sonographic measures was limited [41]. This finding suggests that bowel sounds are not a simple proxy for ultrasound-defined motility and that analytic refinement is required before acoustic monitoring can inform clinical decision-making independently.

### 4.4. Electrogastrography (EGG)

Electrogastrography (EGG) is a noninvasive method that records cutaneous gastric myoelectrical activity to quantify key rhythm domains—bradygastria, normogastria, and tachygastria (Table 3). EGG methodology, analytic pitfalls, and validation considerations have been comprehensively reviewed elsewhere [80]. In neonates, multimodal physiologic acquisition incorporating EGG alongside abdominal NIRS and bowel acoustics has been demonstrated as feasible, supporting bedside implementation in research settings [79].

#### 4.4.1. Potential Role in Predicting Feeding Intolerance and NEC Risk

Current neonatal EGG evidence supports EGG primarily as a marker of gastrointestinal maturation and feeding-related physiology, rather than NEC prediction. Frequency-domain EGG metrics have been evaluated as noninvasive measures of GI maturity in preterm infants [81]. In a longitudinal cohort study, tachygastria burden was quantified over time and related to feeding progression, reinforcing the potential to stratify infants by motility phenotype relevant to feeding tolerance [82]. Recent work using EGG-derived spectral ratio metrics demonstrated that preterm infants with feeding intolerance exhibit attenuated feeding-related gastric myoelectrical responses compared with infants without feeding intolerance, particularly during active feeding periods. Because feeding intolerance can precede NEC in some infants, EGG could help identify infants with vulnerable motility physiology who merit closer imaging surveillance. Together, these findings support EGG as an objective physiologic marker of impaired feeding-related motility [83]. However, it is important to note that direct NEC-predictive validation remains lacking.

#### 4.4.2. Technical Aspects and Practical Considerations

Key barriers to clinical implementation of neonatal EGG include motion artifact, cardiogenic interference, electrode placement variability, and limited analytic standardization across devices [80]. These factors reduce signal reliability and complicate interpretation in individual patients. Advances in signal processing and artifact rejection have enabled more stable long-duration gastric myoelectrical recordings in ambulatory settings, although neonatal validation remains necessary [84]. Interpretation of neonatal EGG is further complicated by ongoing postnatal maturation of gastric electrical activity in preterm infants, underscoring the need for age-specific normative frameworks [85]. At present, EGG should be considered an investigational adjunct within multimodal physiologic monitoring rather than a validated predictive tool for NEC.

## 5. Artificial Intelligence, Imaging, and NEC

Artificial intelligence (AI)-based methods are increasingly being investigated to augment imaging-based diagnosis and risk stratification in NEC (Table 2). Although the current literature remains limited, multiple retrospective studies suggest that AI applied to neonatal imaging, particularly with AXRs, may aid in early diagnosis and prediction of the need for surgical intervention. A range of AI techniques have been explored, including radiomics and deep learning, often in combination with clinical data.

### 5.1. Radiography and Radiomics

Radiomics is a computer-aided imaging approach that extracts large numbers of quantitative features from medical images that are not appreciable by visual inspection alone [86]. These features characterize image intensity, texture, heterogeneity, and shape and, following feature reduction, are commonly incorporated into machine-learning models such as logistic regression, support vector machine, random forest, and gradient boosting. Compared with deep learning, radiomics-based models are generally more interpretable, as individual features are mathematically defined and their relative contributions can be examined.

Lu et al. [87] retrospectively analyzed radiographs from 484 neonates (262 with NEC and 222 without NEC) and developed a radiomics-based model for diagnosing NEC using LASSO feature selection and logistic regression. The model demonstrated moderate performance, with area under the curve (AUC) of 0.82, 0.74, and 0.71 in the training, test, and temporal validation cohorts, respectively. Li et al. [88] evaluated 171 newborns with NEC and developed models to predict the need for surgical intervention using clinical variables, radiomic features, and combined multimodal inputs. While clinical-only and radiomics-only models showed similar performance, a multimodal eXtreme Gradient Boosting (XGBoost) model achieved a very high predictive accuracy (AUC 0.99 in training and 0.96 in testing) and outperformed the clinical-only model.

### 5.2. Radiography and Deep Learning

Deep learning is a subset of AI that uses multi-layered neural networks, most commonly convolutional neural networks (CNNs), to automatically learn discriminative features directly from images [86]. Unlike radiomics, deep learning performs end-to-end image analysis without predefined feature extraction and often achieves high performance with sufficiently large datasets. However, these models are typically less interpretable and are often considered “black boxes.”

Weller et al. [89] applied a deep learning approach to specifically detect NEC-related pneumatosis intestinalis on abdominal radiographs. In this study, 494 neonatal abdominal radiographs (214 with pneumatosis intestinalis and 280 controls) were divided into training, validation, and test sets. Using transfer learning, a ResNet-50 CNN pre-trained on ImageNet achieved an AUC of 0.92 (95% CI: 0.84–0.98) and an accuracy of 87.8%. Model performance in their study was comparable to that of senior surgical residents, suggesting near–expert-level detection of pneumatosis intestinalis.

### 5.3. Combined Radiography, Radiomics, and Deep Learning

More recent studies have focused on integrated AI models that combine both radiomics and deep learning as well as clinical data. Gao et al. [90] developed such multimodal AI models using abdominal radiographs and clinical parameters from infants with suspected NEC, achieving an AUC of 0.93 for NEC diagnosis, outperforming imaging-only and clinical-only models. For predicting the need for surgery, a multimodal approach was again demonstrated by the same authors to have superior performance (AUC 0.94). Similarly, Wu et al. [91] applied a combined deep learning and radiomics framework to abdominal radiographs from two institutions. In external validation, logistic regression and random forest models achieved AUCs of 0.96 and 0.95, respectively, for early NEC detection, outperforming human experts and highlighting the potential value of quantitative imaging approaches for early disease identification.

### 5.4. Ultrasound and Artificial Intelligence

There is increasing evidence that BUS is feasible and should probably play a greater role in the early diagnosis and individualized management of NEC [36,38]. Ultrasound can detect abnormalities such as altered peristalsis, bowel wall thickening, perfusion changes, and intra-abdominal fluid that may precede radiographic findings. Despite this, AI applications using ultrasound in NEC remain limited. Most existing studies have relied on human observer structural ultrasound findings rather than direct image-based analysis. In a retrospective case–control study, Yang et al. [92] evaluated 191 neonates with NEC and matched controls using established ultrasound findings of NEC, clinical variables, demographics, and serologic markers. Among several ML models, an XGBoost model demonstrated the best performance, with an AUC of 0.97 during cross-validation but 0.88 in the validation set, suggesting potential overfitting and the need for external validation. Models using ultrasound or serologic data alone performed substantially worse than multimodal models. Explainability analysis identified bowel peristalsis, C-reactive protein, albumin, bowel wall thickness, and procalcitonin as key predictors.

### 5.5. Limitations and Future Directions of Artificial Intelligence in NEC Imaging

Overall, early evidence suggests that AI applied to imaging may improve diagnosis and risk stratification in NEC, particularly when imaging features are combined with clinical data. However, the current literature is limited by small sample sizes, retrospective designs, and single- or dual-center studies. Larger, prospective, multicenter investigations are needed to establish generalizability and clinical utility. Future AI systems will likely emphasize multimodal integration, incorporating imaging, clinical variables, laboratory data, and potentially emerging biomarkers such as the intestinal microbiome [93]. As these tools mature, attention to interpretability, bias, and workflow integration will be critical to their successful adoption in neonatal care.

## 6. Discussion

Imaging remains central to the diagnosis and monitoring of NEC, but the strengths and limitations of current imaging modalities have shaped a largely reactive diagnostic paradigm. For example, while AXR continues to serve as the cornerstone of initial evaluation because of its availability and integration into established staging frameworks; its hallmark radiographic signs for NEC—pneumatosis intestinalis, portal venous gas, and free intraperitoneal air—reflect already advanced manifestations of intestinal injury. BUS improves upon this reactive paradigm by enabling real-time assessment of bowel wall structure, perfusion, motility, and intra-abdominal fluid—features that complement radiography and may improve risk stratification in equivocal cases. Nevertheless, its clinical impact remains constrained by variability in availability and expertise outside of specialized children’s hospitals. In this context, neonatologist-performed BUS may help expand access to bedside intestinal imaging and facilitate more timely physiologic assessment in the NICU, but its broader adoption will depend on standardized training pathways, quality assurance, and outcome-based validation (Figure 1).

**Figure 3:**
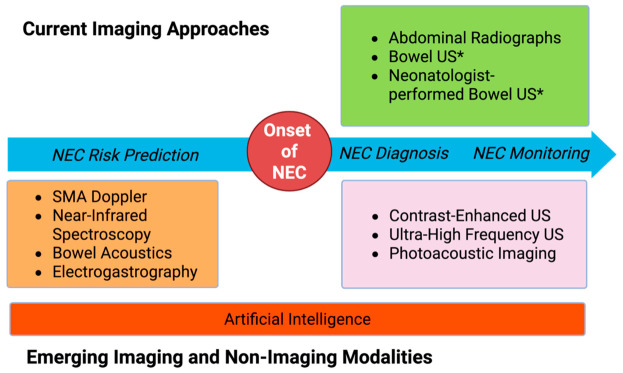
Current and Emerging Approaches to NEC Evaluation. Current imaging strategies (green box) for NEC primarily begin after clinical onset and are used for disease diagnosis and monitoring. Abdominal radiography remains the most widely used initial modality, while bowel ultrasound can provide additional physiologic information when available (*), although access and expertise vary across institutions. Emerging imaging modalities (purple box) may further expand the ability to characterize intestinal injury and support both diagnosis and monitoring. In addition, physiologic monitoring approaches (orange box) aim to detect early alterations in intestinal perfusion, oxygenation, or motility and therefore hold potential for NEC risk prediction before overt clinical disease develops. Artificial intelligence (red box) may further enhance these approaches by integrating imaging and physiologic data to improve risk prediction, diagnostic interpretation, and clinical decision-making.

Emerging imaging modalities and physiologic monitoring tools herald a potential shift from a reactive diagnostic paradigm toward a more proactive, physiology-driven approach focused on early identification of intestinal injury and vulnerability. Techniques such as CEUS, UHFUS, and PAI aim to characterize intestinal microvascular perfusion, oxygenation, and microstructural injury that are not captured by current imaging with AXR and BUS. In parallel, physiologic approaches—including SMA Doppler, NIRS, bowel acoustics, and EGG—seek to identify deviations in intestinal perfusion, oxygenation, or motility that may signal intestinal vulnerability before overt disease develops (Figure 1). Importantly, many of these modalities provide complementary rather than competing information, suggesting that their greatest potential lies in integrated, multimodal assessment rather than isolated application. Artificial intelligence offers an additional opportunity to integrate these imaging and physiologic data, potentially improving diagnostic accuracy, risk stratification, and early detection (Figure 3).

Despite this potential, the current evidence base for many emerging techniques remains limited by small sample sizes, lack of validation, and variable acquisition protocols and reference standards. In addition, physiologic signals are often influenced by confounding factors such as gestational age, hemodynamics, ventilatory support, and feeding status, complicating interpretation and limiting generalizability. As a result, most advanced imaging and monitoring approaches should continue to be viewed as investigational adjuncts rather than replacements for established imaging pathways for NEC.

Taken together, these considerations highlight an evolving trajectory in NEC evaluation from static detection of established intestinal injury toward integrated, physiology-driven approaches capable of identifying intestinal vulnerability earlier in the disease course. Realizing this shift will require not only technological advancement, but also thoughtful clinical integration, standardization, and outcome-focused validation to ensure that emerging tools translate into meaningful improvements in neonatal care.

## Figures and Tables

**Table 1:** Current imaging modalities for diagnosis and monitoring.

Modality	What It Evaluates	Key Strengths	Key Limitations	Clinical Role
AXR	Pneumatosis intestinalis, portal venous gas, pneumoperitoneum, bowel gas pattern	Widely available, rapid acquisition, and embedded in established diagnostic frameworks	Often detects relatively late manifestations of intestinal injury; limited physiologic information; interobserver variability	First-line imaging for suspected NEC and serial monitoring
BUS	Bowel wall thickness and echogenicity, perfusion, peristalsis, and intra-abdominal fluid	Provides real-time assessment of bowel wall without radiation	Operator dependent and not universally available across centers	Adjunct imaging to improve diagnostic confidence and risk stratification
Neonatologist-performed BUS	Same sonographic features as conventional BUS	Immediate bedside availability enabling rapid physiologic assessment	Requires training, credentialing, and institutional support to ensure quality and consistency	Expands access to bowel ultrasound and facilitates timely evaluation when radiology-performed BUS is not available

AXR, abdominal radiography; BUS, bowel ultrasound; NEC, necrotizing enterocolitis.

**Table 2:** Emerging imaging modalities.

Modality	Key Principle	What It Detects	Potential Advantage Over BUS	Current Status
CEUS	Intravenous microbubble contrast agents enhance ultrasound signal	Real-time visualization of bowel wall microvascular perfusion	May detect impaired perfusion earlier than conventional Doppler assessment	Limited clinical use, mainly in specialized centers
UHFUS	Very high-frequency transducers (30–70 MHz) provide markedly increased spatial resolution for superficial tissues	Detailed visualization of bowel wall layers	Enables visualization of bowel wall microanatomy beyond the resolution of conventional US	Early feasibility studies; limited clinical availability
PAI	Hybrid optical-ultrasound technique in which pulsed laser light generates acoustic signals from hemoglobin	Tissue oxygenation saturation and microvascular blood volume	Provides functional assessment of tissue oxygenation and perfusion beyond structural ultrasound findings	Preclinical and early translational studies

CEUS, contrast-enhanced ultrasound; UHFUS, ultra-high-frequency ultrasound; PAI, photoacoustic imaging; BUS, bowel ultrasound.

**Table 3:** Physiologic monitoring for prediction of NEC risk.

Technique	Physiologic Domain	Potential Role	Limitations
SMA Doppler	Mesenteric arterial flow dynamics	May identify impaired intestinal perfusion patterns preceding clinical NEC	Variable predictive value; influenced by feeding status and hemodynamic factors
NIRS	Regional tissue oxygenation (splanchnic rSO_2_)	Continuous noninvasive monitoring of splanchnic oxygenation that may identify impaired intestinal oxygenation patterns preceding clinical disease	Signal variability, motion artifact, and influence of systemic hemodynamics; limited specificity
Bowel acoustics	Bowel sound activity (proxy for intestinal motility)	Detection of alterations in bowel sound patterns that may reflect evolving dysmotility and precede clinical disease	Limited validation and lack of standardized analytic approaches
EGG	Gastric myoelectrical activity	Assessment of gastric electrical rhythms reflecting motility physiology	Technical complexity and limited neonatal clinical validation

## Data Availability

The data presented in this study are available on request from the corresponding author.

## Abbreviations

The following abbreviations are used in this manuscript:

AI	Artificial Intelligence
AUC	Area Under the Curve
AXR	Abdominal Radiography
BUS	Bowel Ultrasound
CPAP	Continuous Positive Airway Pressure
CEUS	Contrast-Enhanced Ultrasound
EGG	Electrogastrography
NEC	Necrotizing Enterocolitis
NICU	Neonatal Intensive Care Unit
NIRS	Near-Infrared Spectroscopy
PAI	Photoacoustic Imaging
POCUS	Point-of-Care Ultrasound
SMA	Superior Mesenteric Artery
UHFUS	Ultra-High-Frequency Ultrasound
